# *rac*-4*H*,5*H*,6*H*,7*H*,8*H*,9*H*,10*H*,11*H*-Cyclo­deca­[*d*][1,2,3]selena­diazole-8-carb­oxy­lic acid

**DOI:** 10.1107/S2414314625002421

**Published:** 2025-03-25

**Authors:** Dieter Schollmeyer, Heiner Detert

**Affiliations:** a University of Mainz, Department of Chemistry, Duesbergweg 10-14, 55099 Mainz, Germany; Katholieke Universiteit Leuven, Belgium

**Keywords:** crystal structure, heterocycle, selenium, medium-sized ring

## Abstract

The chair-shaped mol­ecules in the title crystal, C_11_H_16_N_2_O_2_Se, consist of two pairs of enanti­omers with two conformations (ratio 1:3), differing only in the position of two ring carbon atoms. Two mol­ecules are connected *via* carb­oxy­lic acid hydrogen bridges.

## Abstract 2

The chair-shaped mol­ecules in the crystal consist in two pairs of enanti­omers with two conformations (ratio 1:3), differing only in the position of two ring carbon atoms.

## Graphical abstract

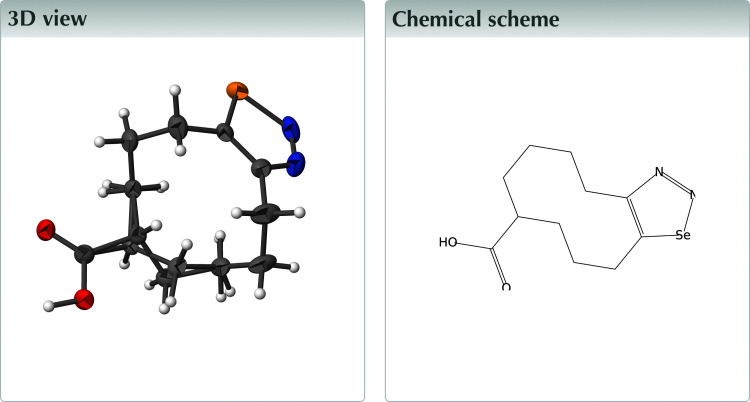

## Structure description

**Figure 1:**
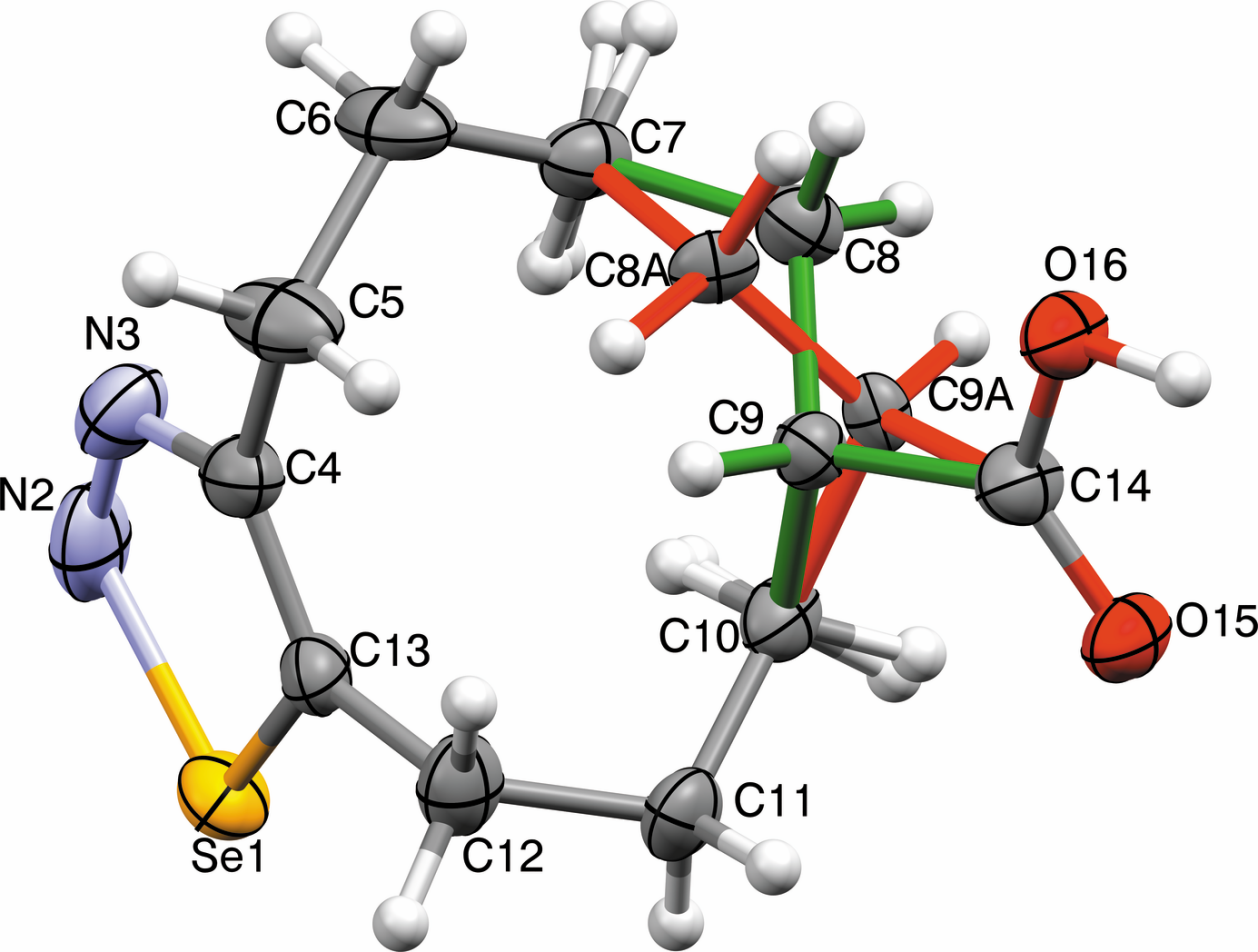
View of the title compound with displacement ellipsoids drawn at the 50% probability level and the minor component shown with red lines (Macrae *et al.*, 2020 ▸).

**Figure 2:**
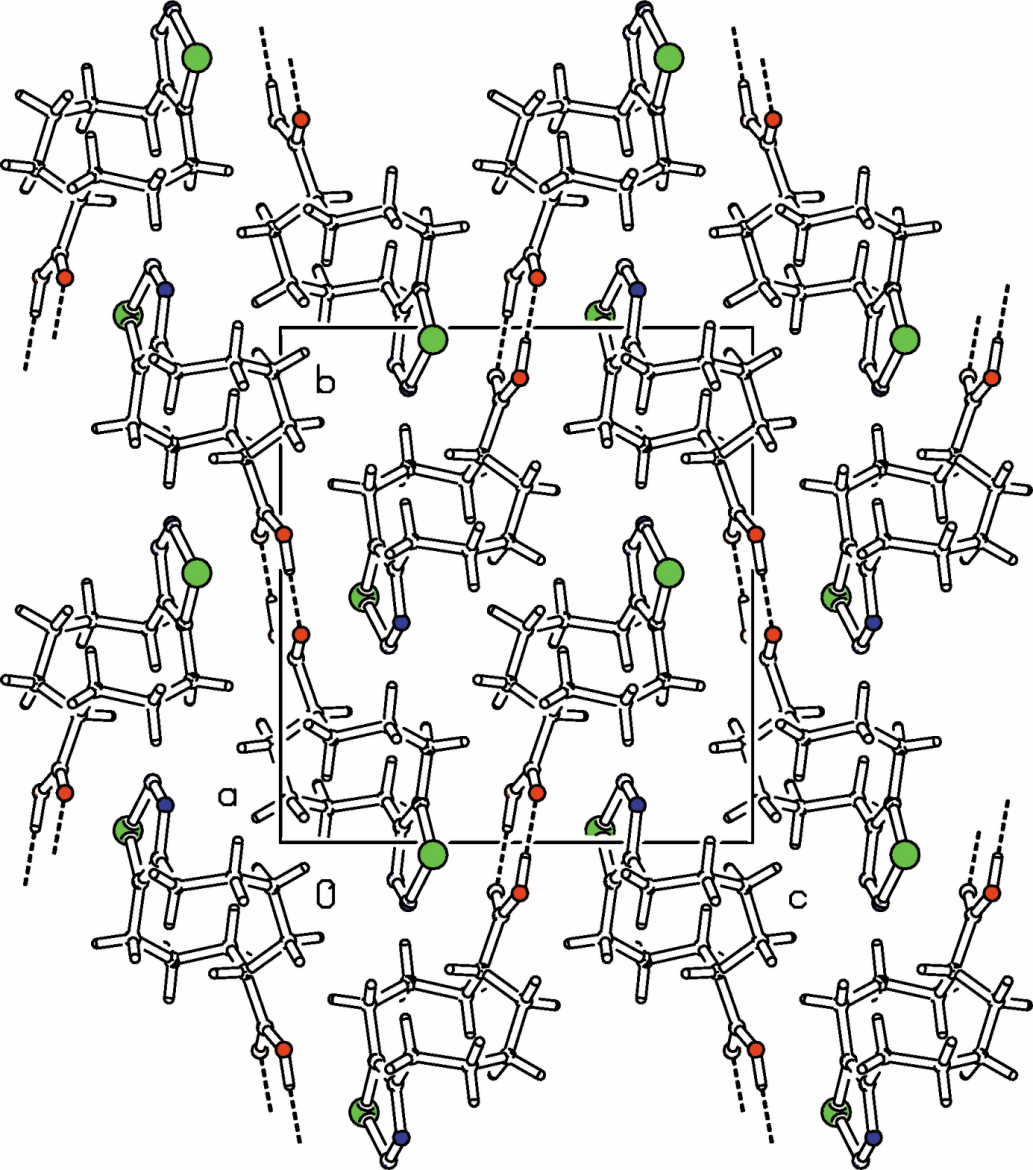
Part of the packing diagram viewed along the *a*-axis direction. Hydrogen bonds are indicated by dashed lines. Only the major disorder component is shown for clarity (Spek, 2009 ▸).

The title compound, C_11_H_16_N_2_O_2_Se, was prepared in a project focusing on transannular cyclizations in medium-sized cyclo­alkynes (Detert *et al.*, 1992 ▸; Detert & Schollmeyer, 2021 ▸; Krämer *et al.*, 2009 ▸). The unit cell contains four chair-shaped mol­ecules, two pairs of enanti­omers with different conformations of the octa­methyl­ene chain in an approximate ratio of 1:3 (Fig. 1 ▸). The selena­diazole ring is planar to within 0.005 (2) Å; the adjacent carbon atoms are slightly below this plane [C5: −0.029 (2) Å, C12: −0.087 (2) Å]. Torsion angles in the octa­methyl­ene tether of the main conformer are C4—C5—C6—C7: −49.4 (3)°, C5—C6—C7—C8: −76.3 (3)°, C6—C7—C8—C9: 70.8 (4)°, C7—C8—C9—C10: 65.5 (5)°, C8—C9—C10—C11: −150.4 (3)°, C9—C10—C11—C12: 62.8 (3)°, and C10—C11—C12—C13: 49.3 (3)°. Interestingly, the positions of most atoms of both conformers are essentially identical, differing only in C8*A* and C9*A* and their attached H atoms. This change provokes a torsion angle C8*A*—C9*A*—C10—C11: −71.5 (9)°. Whereas the C9—H9 bond in the main conformer points in the direction of the selena­diazole plane, this is inverted in the minor conformer. In the extended structure, two mol­ecules are connected *via* H-bridging carb­oxy­lic acids, the distances are O16—H16 = 0.94 (3) Å and O15—H15 = 1.69 (3) Å (Table 1 ▸, Fig. 2 ▸). With an angle of 174 (3)°, the hydrogen bond is slightly bent. The planes of selena­diazole and carb­oxy­lic acid dimer are close to parallel, the dihedral angle being only 7.1 (1)°. The disorder is the consequence of the flexibility of the large ring.

## Synthesis and crystallization

The sample was prepared by G. Krämer (1996 ▸) from 6-hy­droxy­methyl­decan-1-ol (Becker & Chappuis, 1979 ▸) *via* Jones oxidation, formation of the semicarbazone and reaction with selenous acid in 44% overall yield, m.p. 418 K. Crystallization by slow evaporation of a solution in methanol/dichlormethane. ^1^H-NMR (400 MHz, CDCl_3_): 11.0 (*vbs*, 1 H), 3.2 (*m*, 3H), 3.05 (*m*, 1H), 2.62 (*m*, 1 H), 2.22 (*m*, 1 H), 1.90 (*m*, 3 H), 1.80-1.50 (*m*, 3H), 1.30 (*m*, 2 H), 1.03 (*m*, 1 H). ^13^C-NMR (100 MHz, CDCl_3_): 181.6, 159.7, 159.6, 41.5, 29.4, 28.5, 27.4, 25.5, 22.5, 20.0.

## Refinement

Crystal data, data collection and structure refinement details are summarized in Table 2 ▸. Atoms C8 and C9 and their attached H atoms are disordered over two positions [0.759 (8):0.241 (8)].

## Supplementary Material

Crystal structure: contains datablock(s) I, global. DOI: 10.1107/S2414314625002421/vm4065sup1.cif

Structure factors: contains datablock(s) I. DOI: 10.1107/S2414314625002421/vm4065Isup2.hkl

Supporting information file. DOI: 10.1107/S2414314625002421/vm4065Isup3.cml

CCDC reference: 2431691

Additional supporting information:  crystallographic information; 3D view; checkCIF report

## Figures and Tables

**Table 1:** Hydrogen-bond geometry (Å, °)

*D*—H⋯*A*	*D*—H	H⋯*A*	*D*⋯*A*	*D*—H⋯*A*
O16—H16⋯O15^i^	0.94 (3)	1.69 (3)	2.630 (2)	174 (3)

Symmetry code: (i) 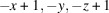.

**Table 2:** Experimental details

Crystal data	
Chemical formula	C_11_H_16_N_2_O_2_Se
*M*_r_	287.22
Crystal system, space group	Monoclinic, *P*2_1_/*n*
Temperature (K)	120
*a*, *b*, *c* (Å)	7.4231 (4), 13.2052 (5), 12.2802 (7)
β (°)	99.725 (4)
*V* (Å^3^)	1186.45 (10)
*Z*	4
Radiation type	Mo *K*α
μ (mm^−1^)	3.15
Crystal size (mm)	0.50 × 0.42 × 0.32

Data collection	
Diffractometer	Stoe Stadivari
Absorption correction	Integration (*X-RED32*; Stoe & Cie, 2020 ▸)
*T*_min_, *T*_max_	0.277, 0.408
No. of measured, independent and observed [*I* > 2σ(*I*)] reflections	7535, 2993, 2458
*R*_int_	0.019
(sin θ/λ)_max_ (Å^−1^)	0.671

Refinement	
*R*[*F*^2^ > 2σ(*F*^2^)], *wR*(*F*^2^), *S*	0.032, 0.085, 1.04
No. of reflections	2993
No. of parameters	168
H-atom treatment	H atoms treated by a mixture of independent and constrained refinement
Δρ_max_, Δρ_min_ (e Å^−3^)	0.61, −0.62

Computer programs: *X-AREAWinXpose*, *Recipe* and *Integrate* (Stoe & Cie, 2020 ▸), *SHELXT2014* (Sheldrick, 2015*a* ▸), *SHELXL2019/3* (Sheldrick, 2015*b* ▸), *PLATON* (Spek, 2009 ▸) and *Mercury* (Macrae *et al.*, 2020 ▸).

## full crystallographic data

### rac-4H,5H,6H,7H,8H,9H,10H,11H-Cyclodeca[d][1,2,3]selenadiazole-8-carboxylic acid Crystal data

**Table:**

C_11_H_16_N_2_O_2_Se	*F*(000) = 584
*M**_r_* = 287.22	*D*_x_ = 1.608 Mg m^−^^3^
Monoclinic, *P*2_1_/*n*	Mo *K*α radiation, λ = 0.71073 Å
*a* = 7.4231 (4) Å	Cell parameters from 13455 reflections
*b* = 13.2052 (5) Å	θ = 2.3–33.3°
*c* = 12.2802 (7) Å	µ = 3.15 mm^−^^1^
β = 99.725 (4)°	*T* = 120 K
*V* = 1186.45 (10) Å^3^	Block, brown
*Z* = 4	0.50 × 0.42 × 0.32 mm

### rac-4H,5H,6H,7H,8H,9H,10H,11H-Cyclodeca[d][1,2,3]selenadiazole-8-carboxylic acid Data collection

**Table:**

Stoe Stadivari diffractometer	2993 independent reflections
Radiation source: Axo Mo	2458 reflections with *I* > 2σ(*I*)
Detector resolution: 13.33 pixels mm^-1^	*R*_int_ = 0.019
rotation method, ω scans	θ_max_ = 28.5°, θ_min_ = 2.3°
Absorption correction: integration (X-Red32; Stoe & Cie, 2020)	*h* = −9→8
*T*_min_ = 0.277, *T*_max_ = 0.408	*k* = −17→11
7535 measured reflections	*l* = −13→16

### rac-4H,5H,6H,7H,8H,9H,10H,11H-Cyclodeca[d][1,2,3]selenadiazole-8-carboxylic acid Refinement

**Table:**

Refinement on *F*^2^	Primary atom site location: dual
Least-squares matrix: full	Hydrogen site location: mixed
*R*[*F*^2^ > 2σ(*F*^2^)] = 0.032	H atoms treated by a mixture of independent and constrained refinement
*wR*(*F*^2^) = 0.085	*w* = 1/[σ^2^(*F*_o_^2^) + (0.0477*P*)^2^ + 0.4785*P*] where *P* = (*F*_o_^2^ + 2*F*_c_^2^)/3
*S* = 1.04	(Δ/σ)_max_ < 0.001
2993 reflections	Δρ_max_ = 0.61 e Å^−^^3^
168 parameters	Δρ_min_ = −0.62 e Å^−^^3^
0 restraints	

### rac-4H,5H,6H,7H,8H,9H,10H,11H-Cyclodeca[d][1,2,3]selenadiazole-8-carboxylic acid Special details

**Table:**

Geometry. All esds (except the esd in the dihedral angle between two l.s. planes) are estimated using the full covariance matrix. The cell esds are taken into account individually in the estimation of esds in distances, angles and torsion angles; correlations between esds in cell parameters are only used when they are defined by crystal symmetry. An approximate (isotropic) treatment of cell esds is used for estimating esds involving l.s. planes.
Refinement. Hydrogen atoms attached to carbons were placed at calculated positions and were refined in the riding-model approximation with C–H = 0.99 Å, and with *U_i_*_so_(H) = 1.2 *U*_eq_(C). Hydrogen atom H16 attached to O16 was refined with an isotropic displacement parameter.

### rac-4H,5H,6H,7H,8H,9H,10H,11H-Cyclodeca[d][1,2,3]selenadiazole-8-carboxylic acid Fractional atomic coordinates and isotropic or equivalent isotropic displacement parameters (Å^2^)

**Table:**

	*x*	*y*	*z*	*U*_iso_*/*U*_eq_	Occ. (<1)
Se1	0.42558 (3)	0.52341 (2)	0.82295 (2)	0.03699 (10)	
N2	0.5805 (3)	0.61713 (15)	0.77083 (18)	0.0457 (5)	
N3	0.7176 (3)	0.57253 (16)	0.74387 (17)	0.0414 (5)	
C4	0.7278 (3)	0.46859 (17)	0.75666 (18)	0.0318 (5)	
C5	0.8880 (3)	0.4149 (2)	0.7219 (2)	0.0459 (6)	
H5A	0.877920	0.341396	0.735378	0.055*	
H5B	1.002410	0.439400	0.767597	0.055*	
C6	0.8980 (3)	0.4323 (2)	0.5990 (2)	0.0451 (6)	
H6A	0.938296	0.502895	0.590196	0.054*	
H6B	0.993277	0.387084	0.578566	0.054*	
C7	0.7242 (3)	0.41505 (16)	0.51877 (19)	0.0330 (5)	
H7A	0.738068	0.451200	0.450044	0.040*	0.759 (8)
H7B	0.626215	0.450426	0.549313	0.040*	0.759 (8)
H7C	0.737146	0.428425	0.441144	0.040*	0.241 (8)
H7D	0.618827	0.452876	0.538069	0.040*	0.241 (8)
C8	0.6499 (5)	0.3088 (2)	0.4837 (3)	0.0323 (9)	0.759 (8)
H8A	0.551274	0.315954	0.419001	0.039*	0.759 (8)
H8B	0.749317	0.268862	0.459877	0.039*	0.759 (8)
C9	0.5750 (5)	0.2491 (2)	0.5736 (4)	0.0253 (8)	0.759 (8)
H9	0.672683	0.245793	0.640491	0.030*	0.759 (8)
C8A	0.7192 (13)	0.2967 (6)	0.5470 (10)	0.024 (3)	0.241 (8)
H8C	0.795335	0.258614	0.502232	0.028*	0.241 (8)
H8D	0.769474	0.285547	0.626060	0.028*	0.241 (8)
C9A	0.5227 (15)	0.2591 (6)	0.5220 (11)	0.020 (2)	0.241 (8)
H9A	0.465069	0.279025	0.445437	0.024*	0.241 (8)
C10	0.4030 (3)	0.29664 (15)	0.60785 (18)	0.0300 (4)	
H10A	0.405841	0.371012	0.598136	0.036*	0.759 (8)
H10B	0.292402	0.270147	0.559887	0.036*	0.759 (8)
H10C	0.419373	0.371049	0.608245	0.036*	0.241 (8)
H10D	0.276217	0.284916	0.569739	0.036*	0.241 (8)
C11	0.3948 (3)	0.27181 (16)	0.72804 (19)	0.0356 (5)	
H11A	0.392631	0.197255	0.736219	0.043*	
H11B	0.278436	0.298372	0.745791	0.043*	
C12	0.5532 (4)	0.31423 (17)	0.8131 (2)	0.0377 (5)	
H12A	0.525003	0.304052	0.888300	0.045*	
H12B	0.665876	0.275742	0.807827	0.045*	
C13	0.5875 (3)	0.42449 (15)	0.79660 (17)	0.0278 (4)	
C14	0.5316 (3)	0.14114 (16)	0.5328 (2)	0.0326 (5)	
O15	0.3937 (2)	0.09629 (11)	0.54373 (14)	0.0366 (4)	
O16	0.6672 (2)	0.09795 (12)	0.49285 (14)	0.0359 (4)	
H16	0.637 (4)	0.029 (2)	0.481 (3)	0.051 (9)*	

### rac-4H,5H,6H,7H,8H,9H,10H,11H-Cyclodeca[d][1,2,3]selenadiazole-8-carboxylic acid Atomic displacement parameters (Å^2^)

**Table:**

	*U*^11^	*U*^22^	*U*^33^	*U*^12^	*U*^13^	*U*^23^
Se1	0.03657 (15)	0.03565 (14)	0.04147 (16)	0.00471 (10)	0.01446 (10)	−0.00838 (10)
N2	0.0629 (15)	0.0245 (9)	0.0463 (12)	−0.0081 (10)	0.0000 (10)	−0.0051 (9)
N3	0.0413 (11)	0.0357 (11)	0.0465 (12)	−0.0110 (9)	0.0056 (9)	0.0039 (9)
C4	0.0290 (11)	0.0375 (12)	0.0290 (11)	−0.0022 (9)	0.0057 (8)	−0.0019 (9)
C5	0.0291 (12)	0.0719 (18)	0.0383 (13)	0.0079 (12)	0.0103 (10)	0.0043 (12)
C6	0.0314 (12)	0.0673 (17)	0.0408 (13)	0.0034 (12)	0.0181 (10)	0.0060 (13)
C7	0.0404 (12)	0.0226 (9)	0.0396 (12)	0.0003 (9)	0.0170 (10)	0.0017 (9)
C8	0.0418 (19)	0.0257 (14)	0.031 (2)	0.0011 (13)	0.0113 (16)	0.0014 (13)
C9	0.0288 (17)	0.0209 (13)	0.026 (2)	0.0001 (12)	0.0051 (14)	0.0003 (13)
C8A	0.026 (5)	0.017 (4)	0.028 (6)	0.005 (3)	0.006 (4)	0.010 (3)
C9A	0.026 (5)	0.014 (4)	0.018 (5)	0.005 (3)	−0.001 (4)	0.002 (4)
C10	0.0288 (10)	0.0206 (9)	0.0424 (12)	0.0009 (8)	0.0115 (9)	−0.0055 (8)
C11	0.0414 (13)	0.0234 (9)	0.0428 (13)	−0.0089 (9)	0.0093 (10)	−0.0023 (9)
C12	0.0459 (14)	0.0249 (10)	0.0408 (13)	−0.0033 (10)	0.0031 (10)	0.0002 (9)
C13	0.0297 (10)	0.0250 (9)	0.0293 (10)	−0.0014 (8)	0.0062 (8)	−0.0069 (8)
C14	0.0360 (12)	0.0235 (9)	0.0415 (12)	0.0014 (9)	0.0157 (9)	−0.0040 (9)
O15	0.0368 (9)	0.0240 (7)	0.0526 (10)	−0.0040 (7)	0.0184 (7)	−0.0080 (7)
O16	0.0399 (9)	0.0220 (7)	0.0501 (10)	−0.0016 (6)	0.0203 (7)	−0.0092 (7)

### rac-4H,5H,6H,7H,8H,9H,10H,11H-Cyclodeca[d][1,2,3]selenadiazole-8-carboxylic acid Geometric parameters (Å, º)

**Table:**

Se1—C13	1.840 (2)	C9—C10	1.544 (4)
Se1—N2	1.874 (2)	C9—H9	1.0000
N2—N3	1.267 (3)	C8A—C9A	1.522 (15)
N3—C4	1.382 (3)	C8A—H8C	0.9900
C4—C13	1.355 (3)	C8A—H8D	0.9900
C4—C5	1.507 (3)	C9A—C14	1.564 (9)
C5—C6	1.540 (3)	C9A—C10	1.570 (9)
C5—H5A	0.9900	C9A—H9A	1.0000
C5—H5B	0.9900	C10—C11	1.523 (3)
C6—C7	1.503 (3)	C10—H10A	0.9900
C6—H6A	0.9900	C10—H10B	0.9900
C6—H6B	0.9900	C10—H10C	0.9900
C7—C8	1.543 (4)	C10—H10D	0.9900
C7—C8A	1.603 (8)	C11—C12	1.540 (3)
C7—H7A	0.9900	C11—H11A	0.9900
C7—H7B	0.9900	C11—H11B	0.9900
C7—H7C	0.9900	C12—C13	1.498 (3)
C7—H7D	0.9900	C12—H12A	0.9900
C8—C9	1.536 (5)	C12—H12B	0.9900
C8—H8A	0.9900	C14—O15	1.210 (3)
C8—H8B	0.9900	C14—O16	1.321 (3)
C9—C14	1.527 (4)	O16—H16	0.94 (3)
			
C13—Se1—N2	87.26 (10)	C7—C8A—H8C	109.8
N3—N2—Se1	110.39 (15)	C9A—C8A—H8D	109.8
N2—N3—C4	117.57 (19)	C7—C8A—H8D	109.8
C13—C4—N3	115.9 (2)	H8C—C8A—H8D	108.3
C13—C4—C5	126.3 (2)	C8A—C9A—C14	106.4 (8)
N3—C4—C5	117.7 (2)	C8A—C9A—C10	113.1 (9)
C4—C5—C6	112.2 (2)	C14—C9A—C10	106.1 (6)
C4—C5—H5A	109.2	C8A—C9A—H9A	110.3
C6—C5—H5A	109.2	C14—C9A—H9A	110.3
C4—C5—H5B	109.2	C10—C9A—H9A	110.3
C6—C5—H5B	109.2	C11—C10—C9	110.3 (2)
H5A—C5—H5B	107.9	C11—C10—C9A	134.7 (5)
C7—C6—C5	116.2 (2)	C11—C10—H10A	109.6
C7—C6—H6A	108.2	C9—C10—H10A	109.6
C5—C6—H6A	108.2	C11—C10—H10B	109.6
C7—C6—H6B	108.2	C9—C10—H10B	109.6
C5—C6—H6B	108.2	H10A—C10—H10B	108.1
H6A—C6—H6B	107.4	C11—C10—H10C	103.5
C6—C7—C8	123.2 (3)	C9A—C10—H10C	103.5
C6—C7—C8A	93.1 (5)	C11—C10—H10D	103.5
C6—C7—H7A	106.5	C9A—C10—H10D	103.5
C8—C7—H7A	106.5	H10C—C10—H10D	105.3
C6—C7—H7B	106.5	C10—C11—C12	115.33 (19)
C8—C7—H7B	106.5	C10—C11—H11A	108.4
H7A—C7—H7B	106.5	C12—C11—H11A	108.4
C6—C7—H7C	113.1	C10—C11—H11B	108.4
C8A—C7—H7C	113.1	C12—C11—H11B	108.4
C6—C7—H7D	113.1	H11A—C11—H11B	107.5
C8A—C7—H7D	113.1	C13—C12—C11	112.92 (19)
H7C—C7—H7D	110.5	C13—C12—H12A	109.0
C9—C8—C7	115.0 (3)	C11—C12—H12A	109.0
C9—C8—H8A	108.5	C13—C12—H12B	109.0
C7—C8—H8A	108.5	C11—C12—H12B	109.0
C9—C8—H8B	108.5	H12A—C12—H12B	107.8
C7—C8—H8B	108.5	C4—C13—C12	128.7 (2)
H8A—C8—H8B	107.5	C4—C13—Se1	108.86 (16)
C14—C9—C8	109.0 (3)	C12—C13—Se1	122.37 (17)
C14—C9—C10	109.3 (2)	O15—C14—O16	122.81 (19)
C8—C9—C10	113.8 (3)	O15—C14—C9	123.8 (2)
C14—C9—H9	108.2	O16—C14—C9	113.1 (2)
C8—C9—H9	108.2	O15—C14—C9A	118.2 (4)
C10—C9—H9	108.2	O16—C14—C9A	115.0 (4)
C9A—C8A—C7	109.3 (8)	C14—O16—H16	107 (2)
C9A—C8A—H8C	109.8		
			
C13—Se1—N2—N3	−0.48 (17)	C9—C10—C11—C12	62.8 (3)
Se1—N2—N3—C4	0.2 (3)	C9A—C10—C11—C12	73.3 (6)
N2—N3—C4—C13	0.4 (3)	C10—C11—C12—C13	49.3 (3)
N2—N3—C4—C5	178.4 (2)	N3—C4—C13—C12	175.9 (2)
C13—C4—C5—C6	119.0 (3)	C5—C4—C13—C12	−1.8 (4)
N3—C4—C5—C6	−58.7 (3)	N3—C4—C13—Se1	−0.8 (2)
C4—C5—C6—C7	−49.4 (3)	C5—C4—C13—Se1	−178.56 (18)
C5—C6—C7—C8	−76.3 (3)	C11—C12—C13—C4	−107.2 (3)
C5—C6—C7—C8A	−64.3 (4)	C11—C12—C13—Se1	69.1 (2)
C6—C7—C8—C9	70.8 (4)	N2—Se1—C13—C4	0.68 (16)
C7—C8—C9—C14	−172.2 (3)	N2—Se1—C13—C12	−176.28 (19)
C7—C8—C9—C10	65.5 (5)	C8—C9—C14—O15	−136.1 (3)
C6—C7—C8A—C9A	154.6 (8)	C10—C9—C14—O15	−11.2 (5)
C7—C8A—C9A—C14	171.0 (5)	C8—C9—C14—O16	49.5 (4)
C7—C8A—C9A—C10	−72.8 (11)	C10—C9—C14—O16	174.5 (2)
C14—C9—C10—C11	87.5 (3)	C8A—C9A—C14—O15	162.0 (7)
C8—C9—C10—C11	−150.4 (3)	C10—C9A—C14—O15	41.2 (9)
C8A—C9A—C10—C11	−71.5 (9)	C8A—C9A—C14—O16	−39.9 (11)
C14—C9A—C10—C11	44.9 (10)	C10—C9A—C14—O16	−160.7 (5)

### rac-4H,5H,6H,7H,8H,9H,10H,11H-Cyclodeca[d][1,2,3]selenadiazole-8-carboxylic acid Hydrogen-bond geometry (Å, º)

**Table:**

*D*—H···*A*	*D*—H	H···*A*	*D*···*A*	*D*—H···*A*
O16—H16···O15^i^	0.94 (3)	1.69 (3)	2.630 (2)	174 (3)

Symmetry code: (i) −*x*+1, −*y*, −*z*+1.
